# Uptake and Function Studies of Maternal Milk-derived MicroRNAs*

**DOI:** 10.1074/jbc.M115.676734

**Published:** 2015-08-03

**Authors:** Alexandra C. Title, Rémy Denzler, Markus Stoffel

**Affiliations:** From the ‡ Institute of Molecular Health Sciences, Eidgenössische Technische Hochschule (ETH) Zurich, Otto-Stern-Weg 7, 8093 Zurich, Switzerland and; the § Faculty of Medicine, University of Zurich, 8091 Zurich, Switzerland

**Keywords:** gene expression, intestine, mammary gland, microRNA (miRNA), nutrition, absorption, milk

## Abstract

MicroRNAs (miRNAs) are important regulators of cell-autonomous gene expression that influence many biological processes. They are also released from cells and are present in virtually all body fluids, including blood, urine, saliva, sweat, and milk. The functional role of nutritionally obtained extracellular miRNAs is controversial, and irrefutable demonstration of exogenous miRNA uptake by cells and canonical miRNA function is still lacking. Here we show that miRNAs are present at high levels in the milk of lactating mice. To investigate intestinal uptake of miRNAs in newborn mice, we employed genetic models in which newborn miR-375 and miR-200c/141 knockout mice received milk from wild-type foster mothers. Analysis of the intestinal epithelium, blood, liver, and spleen revealed no evidence for miRNA uptake. miR-375 levels in hepatocytes were at the limit of detection and remained orders of magnitude below the threshold for target gene regulation (between 1000 and 10,000 copies/cell). Furthermore, our study revealed rapid degradation of milk miRNAs in intestinal fluid. Together, our results indicate a nutritional rather than gene-regulatory role of miRNAs in the milk of newborn mice.

## Teaser

**Background:** It is unclear whether maternal milk microRNAs are taken up by offspring.

**Results:** Milk microRNAs are not taken up into murine offspring tissues or blood but are degraded by the digestive system.

**Conclusion:** It is unlikely that milk microRNAs function through canonical microRNA silencing.

**Significance:** Nutritionally derived microRNAs are unlikely to cross the intestinal barrier and influence gene expression.

## Introduction

MicroRNAs (miRNAs)^2^ comprise an abundant class of non-coding RNAs that are ∼22 nucleotides in length. They can play important regulatory roles in plants, animals, and humans by targeting mRNAs for degradation or translational repression, thereby influencing the output of many protein-coding genes (1). Major functions of miRNAs include helping to maintain a particular differentiation state and to preserve cellular identity in fully developed tissues (2) as well as influencing responses to physiological and pathophysiological stress (3). Additionally, dysregulation of miRNAs has been correlated with various disease states, including metabolic dysfunction and cancer (4, 5). Apart from their cell-autonomous functions, miRNAs are also present in numerous body fluids, including blood, saliva, urine, amniotic fluid, and milk (6). The existence of extracellular miRNAs raises the interesting questions of whether they are released in a regulated fashion and whether circulating miRNAs in mammals act at a distance in intertissue communication (7). Moreover, the concept of miRNA regulation from distant cells within the same organism and, potentially, even from different organisms has led to the question of whether miRNAs from exogenous sources such as food could affect gene expression in human tissues. Although the uptake of nutritionally derived miRNAs was originally demonstrated in 2012 (8), this notion has since been rejected by multiple studies demonstrating that miRNAs originating from dietary sources, crossing the intestinal barrier and regulating gene expression exogenously, is highly unlikely (9–12).

Milk in lactating mothers has been shown to be a particularly rich source of secreted miRNAs. Sequencing and microarray analysis of miRNAs in the milk of various mammalian species has led to the discovery of hundreds of miRNAs in bovine (13–15), porcine (16, 17), rat (18), and human milk (19–22). miRNAs in milk are derived from exosomes and cellular components. The high representation of so-called “immune-related miRNAs” implies that they may play a role in immunity in newborn mammals (14, 15, 17, 18, 22). Furthermore, several studies have suggested that milk miRNAs may survive degradation in the digestive tract because they have been shown to be particularly resistant to harsh treatment such as incubation under acidic conditions and at high temperatures, likely because of their presence in exosomes (14, 15, 17, 22). Therefore, although it may appear as though miRNA transfer from the milk to offspring would contradict the numerous failures to demonstrate the uptake of nutritionally derived miRNAs in adult rodents and humans, it is plausible that same species-derived milk miRNAs encapsulated in exosomes may be taken up in the neonatal period when the intestinal barrier is not fully developed.

Several *in vivo* studies have been performed in an attempt to understand whether milk miRNAs are subject to uptake by offspring. Gu *et al.* (17) have reported that pigs fed only colostrum, as opposed to mature milk, had a higher abundance of colostrum-enriched miRNAs circulating in their serum, inferring that these miRNAs were transferred through the digestive tract of the recipient offspring. A second study (9) reported a dose-dependent increase in plasma miR-29b and miR-200c in human subjects who consumed 0.25–1 liter of milk and suggested target gene regulation in peripheral blood mononuclear cells. Finally, a more recent study has revealed no evidence of miRNA uptake in murine offspring consuming milk overexpressing miR-30b (23). Importantly, all of these studies share an inability to distinguish between endogenous and exogenous copies of the miRNAs in question.

This study was designed to help resolve the issue of whether miRNAs can be taken up from milk and regulate gene expression by canonical miRNA function in tissues of newborn and young mice during the lactation period. To prevent confounding effects of miRNAs that are derived from tissues of suckling offspring, we utilized two different miRNA-deficient mouse strains as a model system, the miR-375 knockout (375KO) mouse and the miR-200c/141 knockout (200cKO) mouse. miR-375 was originally described as pancreas-specific and as an important regulator of insulin secretion and α and β cell mass (24, 25). Subsequently it has also been found to be expressed in a variety of other tissues, to be down-regulated in some cancers (26), and to play a role in immunity (27). miR-200c, a prototypical epithelial miRNA, is a member of the miR-200 family composed of two chromosomal clusters: miR-200a/200b/429 and miR-200c/141 (28). Importantly, both miR-375 and miR-200c have been detected in rat milk whey (18) and have been found to be among the top 10 most expressed miRNAs in porcine milk exosomes (17) and, in the case of miR-200c, in human milk as well (21).

## Experimental Procedures

### 

#### 

##### Animals

375KO and 200cKO mice were generated as described previously on a C57BL/6N background (25, 29). C57BL/6N mice (Janvier) were used for hepatocyte isolation as described below. All animals were kept at 21 °C in a pathogen-free animal facility at the Institute of Molecular Health Sciences at the Eidgenössische Technische Hochschule Zurich on a 12-h light-dark cycle and fed standard laboratory chow and water *ad libitum*.

##### Milk Small RNA Sequencing

Denatured milk was collected from the stomachs of WT offspring aged 2 days (D), 8 days, and 14 days, representing the early, mid, and late stages of lactation, with two replicates each. RNA was extracted, and small RNA sequencing was performed by Fasteris (Geneva, Switzerland). Library preparation consisted of size selection for 18- to 30-nt fragments, followed by 5′ and 3′ adapter ligation and amplification using the TruSeq SBS kit v3 (Illumina). Single reads were then sequenced on an Illumina HiSeq 2500. miRNAs were identified by comparing the first 20 nucleotides of the raw sequence to the known *Mus musculus* miRNA sequences in miRBase (release 20). Relative abundance was calculated as the percentage ratio of “unique miRNA count” over “total miRNA count.” For all analyses, the miRNA abundance of lactation day replicates was averaged. For pairwise comparisons of global miRNA abundance between lactation day samples, miRNAs expressed in only one of the two samples being compared were set as 0% in the non-expressing sample.

##### Experimental Breeding and Tissue Collection

Breedings were set up independently for 375KO and 200cKO strains, following the schematic in Fig. 2*A*. Homozygote breedings were set up to generate WT or KO litters. To generate WT pups receiving KO milk (Group 2) and KO pups receiving WT milk (Group 3), litters were exchanged within 1 day of birth. New pups were wrapped in bedding of the original litter to take up its scent, which was sufficient to induce mothers to care for the new litter. Pups of Groups 1 and 4 (Fig. 2*A*) were left with their respective mothers. At least three litters were produced per pup-genotype/milk-genotype combination to avoid litter-specific effects. Offspring were sacrificed at either D14 or D3 for 375KO breeding and D14 for 200cKO breeding. Blood was collected as described below. Milk was collected from the stomach by incision at the greater curvature and gentle removal of its contents.

##### RNA Extraction and RT-PCR

RNA extraction of tissues (liver and spleen), cells (enterocytes and hepatocytes), and intestinal contents was performed using either TRI Reagent (Sigma) or peqGOLD TriFast (Peqlab) according to the instructions of the manufacturer, with an additional incubation step of 30 min in isopropanol at −20 °C and two washes with 75% EtOH. GlycoBlue coprecipitant (Life Technologies) was used as a carrier.

RNA from milk clots and plasma was isolated using the miRNeasy kit (Qiagen) following the supplementary protocol for purification of RNA from serum or plasma. Samples were spiked with 20 fmol synthetic cel-miR-39 as an internal control for extraction efficiency (adapted from Kroh *et al.* (30)) because of a lack of well established normalization controls for milk or blood. 50 μl of plasma was utilized for D14 pups and 20 μl for D3 pups because of the smaller blood volume collected. Additionally, 240 ng of MS2 RNA (Roche) was added as a carrier. All samples were eluted from the columns in 30 μl of RNase-free water. RNA concentration and purity were measured on a Nanodrop ND-1000 (Thermo Scientific). RNA quality was checked on a Bioanalyzer 2100 (Agilent).

For miRNA quantitative PCR (qPCR), unless stated otherwise, 50 ng of RNA was reverse-transcribed using the TaqMan MicroRNA assay kit (Life Technologies) with primers from miRNA-specific TaqMan small RNA assays (Life Technologies) in a volume of 15 μl (hsa-miR-375, assay 000564; hsa-miR-200a, assay 000502; hsa-miR-200c, assay 002300; hsa-let-7f, assay 000382; hsa-miR-194, assay 000493; hsa-miR-122, assay 002245; cel-miR-39, assay 000200; snoRNA202, assay 001232; hsa-miR-33a, custom assay CS39QON; and hsa-miR-16, assay 000391). qPCR reactions were scaled down to a total volume of 10 μl, with the RT product present in a 1:14 final dilution. qPCR reactions were performed in technical duplicates, the average of which was used for further analysis. Relative miRNA expression was analyzed using the ddCt method, with sno202 as an internal control. For absolute quantification of miRNAs, synthetic miRNAs comprising the mature miRNA sequence (Sigma) were serially diluted in water and used as input for RT-qPCR reactions, generating standard curves against which to compare experimental cycle threshold (Ct) values (mmu-miR-375-3p, 5′-UUUGUUCGUUCGGCUCGCGUGA-3′; mmu-miR-200a-3p, 5′-UAACACUGUCUGGUAACGAUGU-3′; mmu-miR-200c-3p, 5′-UAAUACUGCCGGGUAAUGAUGGA-3′; mmu-let-7f-5p, 5′-UGAGGUAGUAGAUUGUAUAGUU-3′; mmu-miR-194-5p, 5′-UGUAACAGCAACUCCAUGUGGA-3′; mmu-miR-122-5p, 5′-UGGAGUGUGACAAUGGUGUUUG-3′; mmu-miR-33-5p, 5′-GUGCAUUGUAGUUGCAUUGCA-3′; and mmu-miR-16-5p, 5′-UAGCAGCACGUAAAUAUUGGCG-3′). The detection limit of each miRNA assay was designated as the highest Ct value within the linear range of the standard curve generated using serially diluted synthetic miRNAs.

For target gene qPCR, 1 μg of RNA was reverse-transcribed using the high-capacity cDNA reverse transcription kit (Life Technologies). qPCR was performed using 2× KAPA SYBR FAST qPCR Master Mix (Kapa Biosystems) with primers designed to be intron-spanning and gene-specific (*Khsrp*, 5′-GACTCAGGCTGCAAAGTTCA-3′ and 5′-GTGCTCCAGTCAGAGACACG-3′; *Pop4*, 5′-CTTCTGCAGGAGACCAAACA-3′ and 5′-CAATTCAGCTTGGGGATGAC-3′; *Chsy1*, 5′-AACTTTCTCTTCGTGGGAGTCA-3′ and 5′-GGGAATTGTCTTGGACCATGT-3′; *Slc16a2*, 5′-TGAGTATATTCACTGACCGTTTGG-3′ and 5′-TGAAGTAGCGCAGGCTTAGG-3′; *ApoM*, 5′-CCCAGACATGAAAACAGACCT-3′ and 5′-GGGTGTGGTGACCGATTG-3′; and *36b4*, 5′-GCCGTGATGCCCAGGGAAGACA-3′ and 5′-CATCTGCTTGGAGCCCACGTTG-3′). Gene expression was analyzed using the ddCt method with *36b4* as the endogenous control. Reverse transcription reactions were performed on a Mastercycler Gradient (Eppendorf), and qPCR was carried out using a Lightcycler 480 II (Roche).

##### Enterocyte Isolation

Enterocytes were isolated from an upper (jejunum) and lower (ileum) section of the small intestine as well as the colon, as published previously (31), but utilizing a solution of 5 mm EDTA/PBS (Applichem, Gibco) supplemented with Complete protease inhibitor mixture (Roche) and rotation for 1 h at 40 rpm. Cells were disrupted immediately in TRI reagent for subsequent RNA extraction.

##### Blood

Blood was collected using uncoated microhematocrit capillaries (Hirschmann Laborgeräte) in EDTA-containing tubes and centrifuged at 8000 rpm for 10 min to isolate plasma. Following RNA extraction, the protocol proposed by Kroh *et al.* (30) was followed with some modifications. The RT reaction was conducted in 15 μl with a set volume of 2.5 μl of RNA for D14 but 5 μl of RNA for D3 because of the lower input volume of plasma for the D3 RNA extraction. Duplicate qPCR reactions were conducted in a volume of 10 μl with the RT product present in a final dilution of 1:14. Data normalization was performed using cel-miR-39 as described, and normalized Ct values were used for absolute quantification and representation of data as copy number per microliter of plasma.

##### Northern Blotting

14 μg of pooled milk RNA, 40 μg of intestinal content RNA, and 14 μg of mammary tissue RNA (as a positive control) were denatured and separated on a 12% denaturing polyacrylamide gel containing 7.5 m urea (SequaGel, National Diagnostics). Separated RNAs were then transferred onto a Hybond Nx nylon membrane (GE Healthcare Life Sciences) and cross-linked using 1-ethyl-3-(3-dimethylaminopropyl) carbodiimide (Thermo Scientific) as detailed by Pall and Hamilton (32). miR-375 and miR-200c probes were designed as the reverse complement of the mature miRNA sequence (Sigma; miR-375, 5′-TCACGCGAGCCGAACGAACAAA-3′; miR-200c, 5′-TCCATCATTACCCGGCAGTATTA-3′), and 20 pmol was labeled with [γ-^32^P]dATP (PerkinElmer Life Sciences) using T4 polynucleotide kinase (New England Biolabs). Membranes were prehybridized at 50 °C under rotation for 1 h and hybridized with the labeled probe overnight (20 mm Na_2_HPO_4_ (pH 7.2), 7% SDS, 25% 20× SSC, 0.02% albumin, 0.02% polyvinylpyrrolidon K30, 0.02% Ficoll 400, and 0.1 mg/ml sonicated salmon sperm), followed by washing twice with buffer 1 (25% 20× SSC, and 5% SDS) and once with buffer 2 (5% 20× SSC, and 1% SDS) and exposure to a phosphorimaging screen. The signal was detected using the FLA-7000 (FujiFilm). ImageJ (National Institutes of Health) was used for density quantification. miR-375 was measured as target of interest and miR-200c as a positive control for RNA loading.

##### miR-375 Overexpression and Absolute Quantification in Hepatocytes

Hepatocytes were isolated as described previously (33), plated, and infected in quadruplicates with a recombinant adenovirus expressing miR-375 (Ad-miR-375) (24) at 7 multiplicities of infection: 0, 1.5, 3, 6, 12.5, 25, and 50. To ensure equal virus titer per sample, an adenovirus expressing AldoA cDNA with a stop codon at amino acid residue 10 and a mutated miR-122 seed (Ad-AldoA Stop Mut) (33) was used to bring the total virus titer up to a multiplicity of infection of 100, therefore excluding any virus-specific effects. For absolute quantification of miR-375 in hepatocytes, non-infected hepatocyte lysates were spiked with serially diluted synthetic miR-375 as well as miR-33 and miR-16 as controls. Assuming a mean RNA content of 73.5 pg/hepatocyte (33), copy numbers per hepatocyte were calculated and related to a miR-375 standard curve prepared in water, allowing an estimate of baseline copy number per hepatocyte. Multiplication of relative miR-375 expression levels in Ad-miR-375-infected hepatocytes by baseline expression generated values of copy numbers per hepatocyte.

##### miR-375 Target Gene Validation in Hepatocytes and Correlation to miR-375 Expression

Potential target genes were selected by cross-referencing hepatocyte mRNA sequencing data with predicted targets from TargetScan (version 6.2). Four target genes were selected (*Khsrp*, *Pop4*, *Chsy1*, and *Slc16a2*), and their relative expression was evaluated by qPCR in Ad-miR-375-infected hepatocytes. *ApoM* was used as negative control and *36b4* as internal control. Data were plotted against miR-375 copy number adjusted for baseline expression, enabling quantitative correlation between miR-375 and target gene levels in hepatocytes. Expression of the four selected target genes was measured in experimental livers, resulting from 375KO pup swaps.

##### Intestinal Contents

Intestinal contents were collected from D14 375KO pups receiving WT milk and KO milk in the following manner. The small intestine was removed and opened longitudinally, and contents were removed gently. Contents were then either utilized in the “milk clot and intestinal content incubation” or were disrupted immediately in TRI Reagent to inhibit RNase activity.

##### Milk Clot and Intestinal Content Incubation

To determine whether milk miRNAs can be degraded by the mouse digestive system, murine digestive conditions were mimicked by incubating the gastric milk of D14 375KO offspring receiving WT milk with the intestinal contents of 375KO offspring receiving 375KO milk (in each case pooled from eight offspring), in this manner eliminating all sources of miR-375 other than the milk itself. Milk clots and intestinal contents were dounced separately in PBS (Gibco) with an adjusted pH of 5.0 to better mimic murine intestinal pH (34). The homogenized milk clot pool was then spiked with 15 μmol of cel-miR-39 and 500 μl of homogenized milk was combined with 50 μl of homogenized intestinal contents in three replicate tubes, which were then shaken at 37 °C. 50-μl aliquots were collected after 1, 2, 5, 10, 30, 60, and 120 min and disrupted immediately in TRI Reagent. A set volume of 2.5 μl of RNA was used per reverse transcription reaction.

##### Statistical Analysis

Data are represented as mean ± S.E. Statistical significance was determined by unpaired Student's *t* test for pairs of data and analysis of variance followed by Tukey post hoc testing for multiple groups. Offspring data were analyzed in two pairs by Student's *t* test: WT pups with WT milk *versus* WT pups with KO milk, and KO pups with WT milk *versus* KO pups with KO milk. Pairwise comparisons of sequencing data were calculated using Spearman's rank-order correlation. Statistical analysis was carried out using GraphPad Prism (version 5.0b) and R.

## Results

### 

#### 

##### Characterization of miRNAs in Murine Milk

**FIGURE 1.**
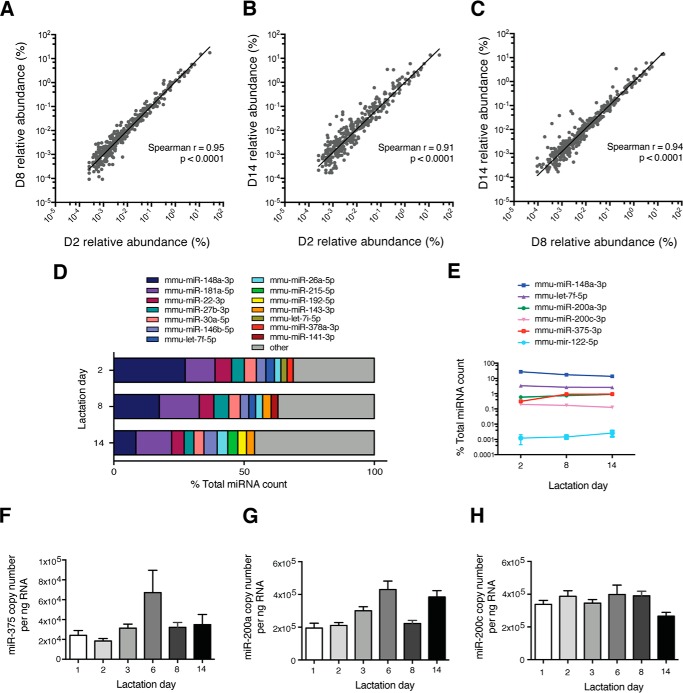
**Characterization of miRNAs in murine milk.** *A–C*, scatter plots depicting the pairwise comparison of global miRNA sequencing profiles in milk at D2, D8, and D14 of lactation (*n* = 2/lactation day): D2 *versus* D8 (*A*), D2 *versus* D14 (*B*), and D8 *versus* D14 (*C*). *D*, the top 10 most highly expressed miRNAs in milk as determined by sequencing per lactation day. *E*, expression profile across lactation days of select miRNAs (*n* = 2). *A–E*, unique miRNAs are expressed as percent of total miRNA count. *F–H*, copy number per nanogram of RNA of miR-375 (*F*), miR-200a (*G)*, and miR-200c (*H*) in WT milk throughout lactation (*n* = 4–6). *E–H*, results are represented as mean ± S.E.

To determine which miRNAs are expressed in whole murine milk, small RNAs (18–30 nucleotides) were sequenced in WT milk clots collected from offspring at days (D) 2, 8, and 14 of lactation. Analysis and comparison of resulting reads to miRBase (release 20) revealed up to 635 miRNAs expressed in a single milk clot sample, with an average of 506 miRNAs/sample. Interestingly, the miRNA profile for different lactation days was similar, with the same seven miRNAs dominating each lactation day (miR-148a-3p, miR-181a-5p, miR-22-3p, miR-27b-3p, miR-30a-5p, miR-146b-5p, and miR-26a-5p) (Fig. 1, *A–D*).

miR-375, designated as the main focus of this study because of its identity as a single-locus miRNA, and miR-200, selected as a secondary example of a milk miRNA, revealed intermediate expression in milk derived from the stomachs of WT mice (Fig. 1*E*). To further examine expression patterns throughout lactation, miR-375 and two representative members of the miR-200 family, miR-200a and miR-200c, were absolutely quantified in milk from pups ranging in age from day 1–14 of lactation. miR-375 was expressed at an average of 4 × 10^4^ copies/ng of RNA (Fig. 1*F*), whereas miR-200a and miR-200c were expressed about 10-fold higher (Fig. 1, *G* and *H*). Furthermore, although there was some variation in miRNA expression within and between lactation days, the copy number always remained within the same order of magnitude. We therefore selected D14 as the main focus for this study, with D3 included for some experiments to account for any potential age-dependent and developmental differences that may affect milk miRNA uptake.

**FIGURE 2.**
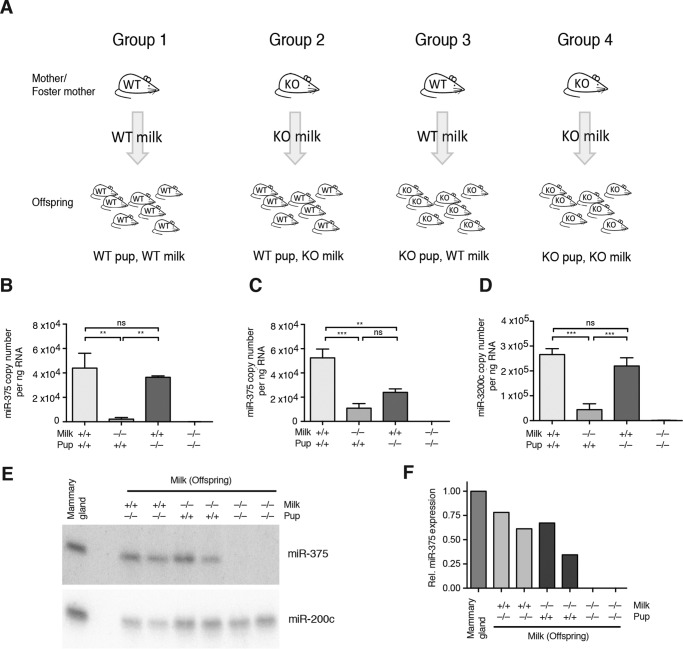
**Offspring exchange breeding scheme and analysis.** *A*, breeding scheme used to generate WT pups receiving WT milk, WT pups receiving KO milk, KO pups receiving WT milk, and KO pups receiving KO milk. Pups were given to foster mothers within 1 day of birth. The breeding scheme was carried out using both 375KO and 200cKO mouse models. *B* and *C*, miR-375 copy number per nanogram of RNA in D14 gastric milk (*B*) and D3 gastric milk (*C*) from pups of the miR-375 pup exchange (*n* = 4). *D*, miR-200c copy number per nanogram of RNA in D14 gastric milk from pups of the miR-200c pup exchange (*n* = 4). *E*, Northern blot analysis of miR-375 expression in D14 milk (14 μg of RNA pooled from three to four pups), with mammary tissue (14 μg of RNA) used as the positive control. miR-200c expression was measured as RNA loading control. *F*, density quantification of miR-375 Northern blot analysis normalized to the mammary gland. *Rel*, relative. *B–D*, results are represented as mean ± S.E. *, *p* ≤ 0.05; **, *p* < 0.01; ***, *p* < 0.001; *ns*, *p* > 0.05.

For the miR-375 portion of this study, offspring of the WT and 375KO genotypes were generated by setting up appropriate matings, and litters were exchanged immediately after birth to generate the following four study groups: WT pups receiving WT milk (WT pup, WT milk), WT pups receiving KO milk (WT pup, KO milk), KO pups receiving WT milk (KO pup, WT milk), and KO pups receiving KO milk (KO pup, KO milk) (Fig. 2*A*). miR-375 levels measured in milk from the stomachs of these offspring revealed a basal contribution of miR-375 from offspring tissues, as evidenced by the presence of miR-375 in the “gastric” milk from WT pups that were nurtured by 375KO mothers (Fig. 2, *B* and *C*). The source of miR-375 in WT pups drinking milk from KO foster mothers likely comes from epithelial cells of the stomach because miR-375 is highly expressed in this tissue (data not shown). Interestingly, this contamination was of greater importance in D3 than in D14 samples, perhaps because of smaller milk clot size and, therefore, a greater surface-to-volume ratio, although some variation in D14 was apparent, as demonstrated by the relatively high miR-375 expression detected in other WT pup, 375KO milk samples by Northern blot analysis (Fig. 2, *E* and *F*). Nevertheless, the data show that, for D14 milk, the majority of miR-375 comes from the milk itself because there was no significant difference between WT pups and 375KO pups receiving WT milk (Fig. 2*B*). Similar results were obtained from an analogous experiment utilizing 200cKO mice (Fig. 2*D*). These comparisons reveal that miR-375 and miR-200c are genuinely highly expressed in milk clots and are not solely the result of contaminating cells or secretions.

##### No Evidence of miRNA Uptake from Milk into Pup Tissues and Blood

**FIGURE 3.**
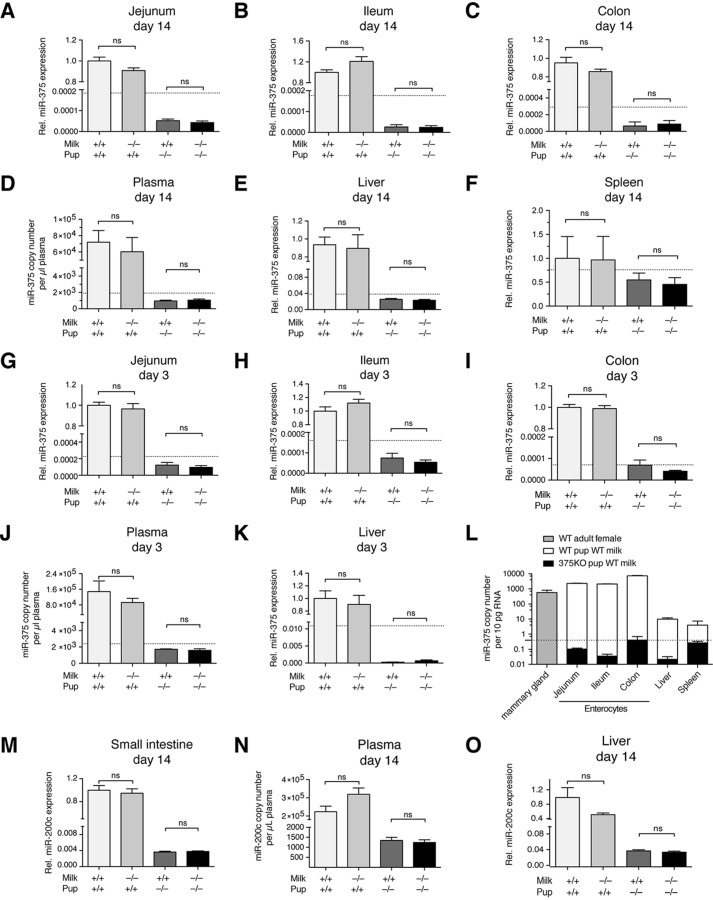
**miR-375 and miR-200c are not taken up into offspring tissues or blood.** *A–K*, relative (*Rel*) miR-375 expression or copy number per microliter of plasma in D14 upper enterocytes (jejunum) (WT pups, *n* = 8; KO pups, *n* = 6) (*A*), D14 lower enterocytes (ileum, *n* = 8) (*B*), D14 colon enterocytes (*n* = 8) (*C*), D14 plasma (WT pups, *n* = 10; KO pups, *n* = 7) (*D*), D14 liver (*n* = 14) (*E*), D14 spleen (*n* = 9) (*F*), D3 jejunum (WT pups, *n* = 8; KO pups, *n* = 6) (*G*), D3 ileum (WT pups, *n* = 8; KO pups, *n* = 6) (*H*), D3 colon enterocytes (WT pups, *n* = 8; KO pups, *n* = 6) (*I*), D3 plasma (*n* = 6–8) (*J*), and D3 liver (WT pups, *n* = 14; KO pups, *n* = 8) (*K*). *L*, tissue panel comparing endogenous miR-375 level/10 pg of RNA (roughly one cell), represented by WT pups receiving WT milk, and exogenous miR-375 level, represented by 375KO pups receiving WT milk (*n* = 6–10). *M–O*, relative miR-200c expression or copy number per microliter of plasma in D14 small intestine enterocytes (*n* = 20) (*M*), D14 plasma (*n* = 8) (*N*), and D14 liver (*n* = 10) (*O*). Relative expression was calculated using the ddCt method with sno202 as an internal normalizer and setting WT pup WT milk as 1. Copy number per microliter of plasma was normalized to spiked-in cel-miR-39. The *dotted lines* represent the detection limit of the qPCR. Values represent mean ± S.E. *, *p* ≤ 0.05; **, *p* < 0.01; ***, *p* < 0.001; *ns*, *p* > 0.05.

To determine whether miRNAs are taken up from maternal milk into offspring tissues, miR-375 expression was evaluated at several potential levels of uptake in D14 offspring from the study groups described previously. The absorptive cells of the intestinal epithelium were deemed the first potential site of uptake. miR-375 expression in isolated enterocytes from an upper portion (jejunum) and lower portion (ileum) of the small intestine as well as the colon revealed no evidence of uptake because there was no measurable increase in miR-375 levels in enterocytes of 375KO pups receiving WT milk compared with 375KO milk (Fig. 3, *A–C*). Furthermore, miR-375 uptake was also undetectable in gastric epithelium (data not shown). The plasma fraction of blood was analyzed as the next possible compartment of miRNA uptake, again revealing no difference in miR-375 expression between 375KO pups receiving WT or 375KO milk (Fig. 3*D*). Further analysis of downstream tissues, including liver and spleen, also provided no evidence of uptake (Fig. 3, *E* and *F*). This lack of miRNA absorption via the intestine was corroborated further in enterocytes, plasma, and livers of D3 pups (Fig. 3, *G–K*), implying that younger offspring age does not affect uptake efficiency. Importantly, the miR-375 copy number across all tissues and plasma samples in 375KO pups, regardless of milk, was always at or below the detection limit of qPCR. Assuming the average cell to contain ∼10 pg of RNA (11), this represents less than 1 copy/cell, suggesting any potential uptake to be negligible at most (Fig. 3*L*). Finally, increasing RNA input into the RT-qPCR reaction from 50 to 300 ng did not significantly decrease Ct values in 375KO pups, suggesting that the measured miRNA levels are genuinely extremely low (data not shown).

To further establish whether other miRNAs may be taken up from milk, D14 enterocytes, plasma, and liver samples were analyzed for miR-200c expression in the analogous 200cKO experiments (Fig. 3, *M–O*). Again, there was no evidence of uptake of miR-200c from milk despite its 10-fold higher levels in milk compared with miR-375 (Fig. 1*H*). Determination of a qPCR detection limit was not instructive in this case because of cross-detection of other miR-200 family members, although the lack of change between 200cKO pups receiving WT milk *versus* 200cKO milk suggests a negligible uptake.

##### Insufficient miR-375 Copy Numbers in the Liver to Induce Target Gene Engagement

**FIGURE 4.**
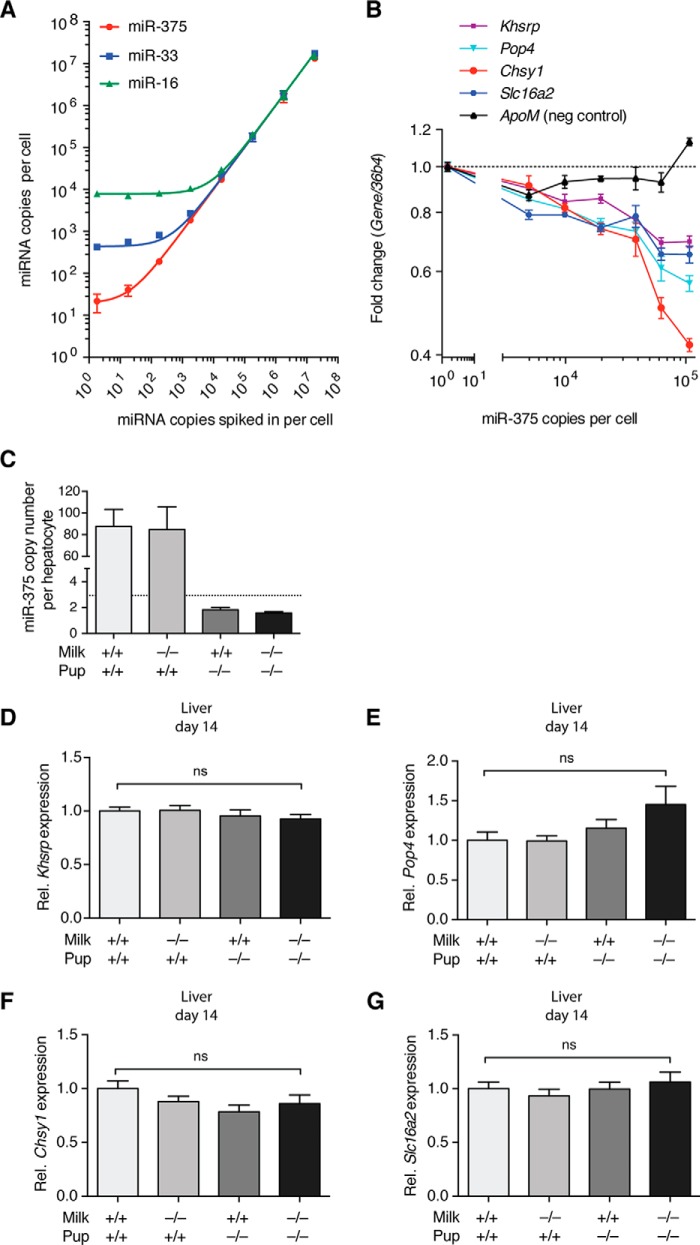
**Evaluation of miR-375 target gene regulation using hepatocytes as a model.** *A*, standard curve built by spiking non-infected hepatocytes with serially diluted synthetic miR-375 (*n* = 3). The copy number detected was on the basis of a standard curve prepared in water. miR-33 and miR-16 were used as controls for linearity. *B*, miR-375 target gene fold-change relative to miR-375 copy number in Ad-miR-375-infected hepatocytes. miR-375 copies per cell represent detected copies in virus-infected hepatocytes of a multiplicity of infection of 0–50 (*n* = 4) on the basis of the standard curve in *A*, with relative target gene expression normalized to a multiplicity of infection of 0. *ApoM* was used as a negative (*neg*) control. *C*, the estimated copy number of miR-375 in D14 offspring liver cells calculated following the assumption that one hepatocyte contains 73.5 pg of RNA (*n* = 14). The *dotted line* represents the detection limit of the qPCR. *D–G*, expression of miR-375 targets *Khsrp* (*D*), *Pop4 (E*), *Chsy1 (F*), and *Slc16a2* (*G*) in D14 offspring livers from the miR-375 pup exchange (*n* = 17–19). Relative target gene expression was calculated using the ddCt method, with *36b4* as an internal normalizer and WT milk WT pup set as 1. Results represent mean ± S.E. *, *p* ≤ 0.05; **, *p* < 0.01; ***, *p* < 0.001; *ns*, *p* > 0.05.

To quantitatively assess the copy number of miR-375 necessary for target gene repression, we used primary hepatocytes that were infected at increasing multiplicities of infection of Ad-miR-375 as a model system. A standard curve was generated via qPCR, comparing synthetic miR-375 copies spiked into hepatocyte lysates with miR-375 copies detected, revealing a baseline level of ∼20 copies of miR-375/cell. miR-33 and miR-16 were used as controls for linearity (Fig. 4*A*). Four predicted miR-375 target genes, *Khsrp*, *Pop4*, *Chsy1*, and *Slc16a2*, revealed dose-dependent repression with increasing miR-375 copy number in infected hepatocytes (Fig. 4*B*). Importantly, this repression only began to be substantial at ∼10^4^ copies of miR-375/cell. Assuming that one hepatocyte contains roughly 73.5 pg of RNA (33), an estimation of copy number per liver cell yielded less than 3 copies/cell in D14 375KO pups receiving WT milk (Fig. 4*C*). This clearly indicates that, even if minimal miRNA uptake from milk were to occur, perhaps undetectable via qPCR, then it would not be sufficient to enable miR-375 to carry out its canonical role of repressing gene expression. Finally, to confirm this hypothesis, we measured the four validated target genes in D14 offspring liver samples. As expected due to unaltered miR-375 copy numbers, expression levels remained unchanged for all targets, suggesting insufficient uptake of miR-375 to have any functional output in the liver (Fig. 4, *D–G*).

##### Degradation of Milk-derived miRNAs in the Intestine

**FIGURE 5.**
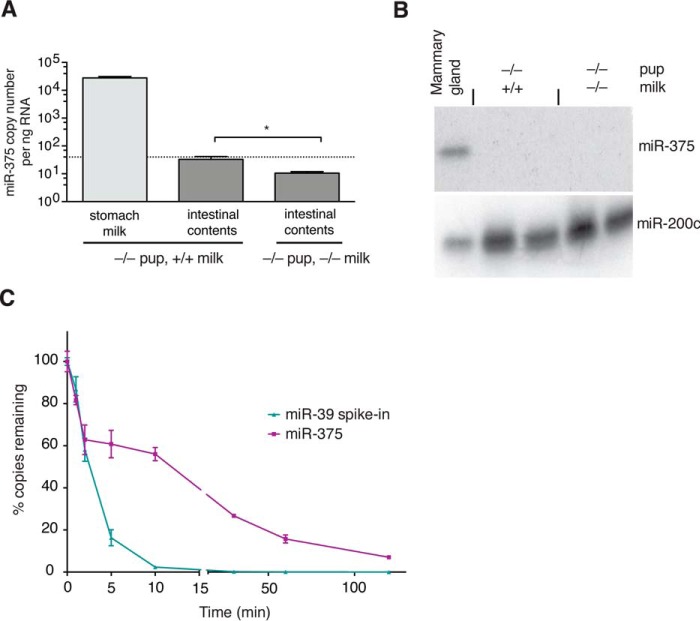
**miR-375 is virtually absent from intestinal contents.** *A*, miR-375 copy number in stomach milk and downstream intestinal contents of D14 375KO offspring (milk, *n* = 8; intestinal contents, *n* = 12). The *dotted line* represents the detection limit of the qPCR. *B*, miR-375 Northern blot analysis of D14 intestinal contents (40 μg) with adult mammary tissue (14 μg) used as a positive control. miR-200c expression was used as an RNA loading control. *C*, WT milk from the stomachs of D14 375KO pups was incubated with the intestinal contents of 375KO pups at 37 °C on a shaker, with equal-volume aliquots collected at specific time points (*n* = 3). Synthetic cel-miR-39 was spiked in as an exogenous control. qPCR absolute quantification was used to determine the percentage of copies remaining relative to the starting material (at time 0). Results represent mean ± S.E.

To determine the fate of milk miRNAs downstream of the stomach, we next chose to analyze the intestinal contents of 375KO pups receiving WT milk. Interestingly, we measured a profound decrease in miR-375 levels in the intestinal contents of these pups relative to the milk clot from the stomach, resulting in only a negligible miR-375 copy number remaining (Fig. 5*A*). This was confirmed further by Northern blot analysis, in which 40 μg of RNA from intestinal contents was loaded to maximize the potential to detect any miR-375 copies (Fig. 5*B*). Such a down-regulation could be explained either via uptake or via degradation. Because there was no evidence of uptake, we proceeded to determine whether milk miRNAs may in fact be subject to degradation. Incubation of milk clots from 375KO pups receiving WT milk with intestinal contents from 375KO pups receiving 375KO milk, assumed to contain intestinal and pancreatic digestive enzymes as well as bile, revealed a time-dependent decrease in miR-375 expression, suggesting that milk miRNAs may, in fact, be degraded by the digestive system (Fig. 5*C*). Importantly, the choice of WT milk from 375KO pups eliminated any potential contribution of miR-375 copies by pup cells. Interestingly, spiked-in cel-miR-39 was degraded more rapidly, suggesting that milk miRNAs do exhibit a certain degree of resistance, perhaps because of their presence in exosomes. Nevertheless, less than 10% of miR-375 copies remained after 2 h of incubation, suggesting that, under physiological digestive conditions, exosomes can be disrupted effectively, thereby subjecting milk-derived miRNAs to enzymatic degradation.

## Discussion

Unambiguous assessment of exogenous miRNA uptake is frequently complicated by the inability to distinguish between exogenous and endogenous copies of the miRNA in question, whether because of same-species origin or a high degree of interspecies conservation in the miRNA sequence. This issue lessens confidence in the claims made by Baier *et al.* (9) that an increase in miR-200c and miR-29b following human consumption of bovine milk is solely due to uptake from the milk because there is exact sequence homology between the bovine and human forms of both miRNAs. Furthermore, the use of an overexpression murine model to study milk miRNA uptake (23) in which WT pups received either WT or miR-30b-overexpressing milk does not account for possible variations in endogenous miR-30b levels and its potential to confound the interpretation of changes in miR-30b expression in the pups. To circumvent this issue and determine unambiguously whether miRNAs derived from maternal milk are taken up by offspring tissues, we designed a clear study using murine KO models to generate KO offspring receiving WT milk, therefore bypassing the need to distinguish endogenous copies. In this study, we chose to concentrate on miR-375 because of its robust expression in murine milk as well as its unique sequence, enabling its unequivocal identification. As an additional example of murine milk miRNA, we selected miR-200c, expressed roughly 10-fold higher than miR-375 in murine milk. miR-200c is a member of a miRNA family composed of five members that are divided into two genomic clusters, miR-141/200c and miR-200a/b/429. Because of infertility of the global miR-200KO mouse (35), we focused on the miR-141/200c KO model. Although high sequence similarity between miR-200 family members (28) led to less reliable quantification of miR-200c, analysis of this second miRNA nevertheless provided additional insights into the behavior of milk miRNAs in general.

Sequencing of small RNAs in milk clots of murine origin revealed the presence of several hundred miRNA species per milk clot whose relative expression did not vary considerably throughout lactation. An overlap of multiple miRNAs between the top 10 highest expressed miRNAs in murine, porcine (17), and human milk (21, 22) suggests evolutionarily conserved expression. Several studies analyzing miRNAs in milk emphasize enrichments in “immune-related” miRNAs such as miR-146b, miR-27b, and the miR-200 family (14, 17, 18, 22), which were also among the more highly expressed miRNAs in murine milk. Although a common conclusion is that milk miRNAs are therefore involved in immune function of the offspring, it should be considered that these could, in fact, be of cellular origin because milk contains mammary gland-derived leukocytes and epithelial cells (36). Finally, our focus on whole murine milk clots enabled the analysis of miRNAs derived from all milk fractions (*i.e.* whey and lipids) and represented exactly what was consumed by the mouse pup. It should be noted, however, that a certain degree of pup-derived cellular contamination was likely.

Systematic analysis of miR-375 and miR-200c expression in pup tissues of KO pups receiving WT milk provided no evidence of miRNA uptake from milk. Intestinal epithelium was identified as the first compartment of dietary miRNA absorption, but no increase in miRNA expression in pups fed WT rather than KO milk was detected. Several mechanisms of miRNA transfer across the intestine have been proposed, including a miRNA-specific transporter, vesicle-mediated transcytosis, or intercellular permeability (37, 38). Both rapid transcytosis and intercellular permeability could explain a lack of detection within enterocytes, suggesting, rather, that miRNAs might be identifiable in the blood. miR-375 and miR-200c analysis of pup plasma, however, revealed no further indication of miRNA uptake. Finally, analysis of several downstream organs exhibiting a profound ability for endocytosis and phagocytosis, such as the liver and spleen, respectively, confirmed a lack of miRNA uptake from milk.

In our study, we chose to focus on D14 of lactation because of the persisting expression of both miR-375 and miR-200c in milk at this later lactation day. The immature neonatal intestine has been shown to behave differently from the mature intestine, demonstrating, for example, increased paracellular permeability (39) and transcytosis of macromolecules (40), whereas maturation is only finalized upon weaning. We also included some offspring from D3 of lactation to account for any developmental changes that might occur gradually. Despite the potential for increased uptake of milk miRNAs, however, we again detected no evidence of such an occurrence.

Our data are in concordance with the results of Laubier *et al.* (23), who found that feeding WT pups with milk overexpressing miR-30b did not increase miR-30b expression in pup blood or tissues. Our study accentuates this lack of uptake even further because the miR-375 copy number difference between WT milk and 375KO milk is more than 1000-fold (a difference of 10 Ct cycles), as opposed to a 31-fold copy number difference between WT milk and miR-30b-overexpressing gastric milk.

The majority of studies on dietary uptake of miRNAs have focused on non-dairy food. Although the initial work by Zhang *et al.* (8) has demonstrated an up-regulation of ∼1000 copies miR-168a per microliter of serum and a 2.5-fold increase in miR-168a expression in the livers of mice following a diet of fresh rice, further studies on plant- and lard-derived miRNAs revealed only a negligible uptake into plasma or tissues, in the range of less than one copy per cell (11). These data are in agreement with our results because we also found less than one copy per cell of miR-375 in tissues of 375KO pups receiving WT milk, following the assumption that the average cell has 10 pg of RNA. Of note, the miRNA copy number in the stomach contents of avocado-fed mice (≈1 × 10^7^ copies of miR-156a and miR-168a) was in a similar range as that of milk-fed pups in our study (estimated at 2.5 × 10^8^ copies of miR-375), assuming the average D14 milk clot to have a mass of 100 mg with 70 ng of RNA/mg. Importantly, quantification of hepatic target gene repression with increasing miR-375 expression implies that such a low copy number would not be sufficient for canonical miRNA function.

Our results are in stark contrast with the conclusion drawn by Baier *et al.* (9) that the up-regulation of 1.5 × 10^5^ copies of miR-200c measured in human plasma following consumption of 250 ml of bovine milk is derived directly from the milk. The authors measured 680 pmol/liter (4 × 10^8^ copies/μl) miR-200c in bovine milk. 250 ml of milk therefore amounts to 1 × 10^14^ copies of miR-200c or 1 × 10^9^ copies/g of body weight of a 75-kg human adult. Following the conservative assumption that the average D14 pup has a stomach volume of 50 μl (41), we can estimate that murine milk contains ≈4 × 10^7^ copies of miR-200c/μl, implying that, for an equivalent copy number consumed per gram of body weight, an 8-g mouse would need to consume 200 μl of milk. This is likely feasible within a few hours because it is estimated that a pup suckles 20% of its body weight in 1 day (1.6 ml for an 8-g pup) (23). Therefore, despite a similar intake of milk-derived miR-200c, we do not detect uptake as reported by Baier *et al.* (9), suggesting that the increase in miR-200c expression they measure may be endogenous (42).

Our final observation that only few copies of miR-375 remain in the contents of the small intestine is important in understanding the fate of milk miRNAs. Although the average miR-375 Ct value in the intestinal contents of 375KO pups receiving WT milk was slightly above the detection limit, several individual pups presented values below, which was not the case for 375KO pups receiving 375KO milk. This suggests that a low number of miR-375 copies may survive the digestive tract but not in a consistent manner. We further corroborated this observation via Northern blot analysis, using 40 μg of intestinal content RNA to maximize potential of detection. Again, only a negligible signal was detected. Incubation of WT milk clots with 375KO intestinal contents, designed to mimic the murine digestive system, revealed that milk miRNAs can, in fact, be degraded under physiological conditions, providing an explanation for this severe decline in copy number in the small intestine. Interestingly, spiked-in cel-miR-39 was degraded at a faster rate, implying that the envelopment in exosomes as described previously (15, 17, 19) may, in fact, protect milk miRNAs from RNases to a certain extent. Several other studies were performed in which milk was subjected to various degradative conditions, including incubation at high temperature or under acidic conditions. Although some studies did report some degradation (17, 22), this was never to the extent that we detected in as short of a time frame. In fact, many studies detected no degradation at all (14, 15). It is likely that this difference is due to our use of physiological digestive conditions, which better mimic genuine intestinal conditions through inclusion of enzymatic activity as well as bile acids rather than a single variable such as high temperature.

In summary, systematic analysis of miRNA-KO pups fed WT milk has revealed an absence of detectable miRNA uptake from milk despite the presence of high copy numbers in milk. Although it remains possible that a small level of uptake does occur and that our methods are simply not sensitive enough to detect it, it is unlikely that such a low copy number would be sufficient for canonical miRNA function. It is therefore most plausible that milk miRNAs serve primarily as a nutritional source of nucleic acids for the rapidly growing offspring.

## Author Contributions

A. C. T. and M. S. designed the study, interpreted results, and wrote the manuscript. A. C. T. performed the majority of experiments. R. D. performed hepatocyte isolation and virus infection and provided assistance with the generation of Fig. 4. All authors reviewed the results and approved the final version of the manuscript.

## Footnotes

2 The abbreviations used are:

miRNA: microRNA
D: day
qPCR: quantitative PCR
Ct: cycle threshold
Ad: adenovirus.
